# An adaptive and versatile method to quantitate and characterize collective cell migration behaviors on complex surfaces

**DOI:** 10.3389/fcell.2023.1106653

**Published:** 2023-01-26

**Authors:** Kristen E. Loesel, Harrison L. Hiraki, Brendon M. Baker, Carole A. Parent

**Affiliations:** ^1^ Cancer Biology Graduate Program, University of Michigan Medical School, Ann Arbor, MI, United States; ^2^ Rogel Cancer Center, University of Michigan Medical School, Ann Arbor, MI, United States; ^3^ Department of Biomedical Engineering, University of Michigan Medical School, Ann Arbor, MI, United States; ^4^ Department of Pharmacology, University of Michigan Medical School, Ann Arbor, MI, United States; ^5^ Department of Cell and Developmental Biology, University of Michigan Medical School, Ann Arbor, MI, United States; ^6^ Life Sciences Institute, University of Michigan, Ann Arbor, MI, United States

**Keywords:** cell migration, contact guidance, electrospinning, fluorescence microscopy, image analysis

## Abstract

Collective cell migration is critical for proper embryonic development, wound healing, and cancer cell invasion. However, much of our knowledge of cell migration has been performed using flat surfaces that lack topographical features and do not recapitulate the complex fibrous architecture of the extracellular matrix (ECM). The recent availability of synthetic fibrous networks designed to mimic *in vivo* ECM has been key to identify the topological features that dictate cell migration patterns as well as to determine the underlying mechanisms that regulate topography-sensing. Recent studies have underscored the prevalence of collective cell migration during cancer invasion, and these observations present a compelling need to understand the mechanisms controlling contact guidance within migratory, multicellular groups. Therefore, we designed an integrated migration analysis platform combining tunable electrospun fibers that recapitulate aspects of the biophysical properties of the ECM, and computational approaches to investigate collective cell migration. To quantitatively assess migration as a function of matrix topography, we developed an automated MATLAB code that quantifies cell migration dynamics, including speed, directionality, and the number of detached cells. This platform enables live cell imaging while providing enough cells for biochemical, proteomic, and genomic analyses, making our system highly adaptable to multiple experimental investigations.

## Introduction

The extracellular matrix (ECM), the non-cellular component of a tissue, acts not only as a supportive cellular scaffold but also provides biochemical and biophysical cues to cells (Walma and Yamada, 2020). ECM composition varies greatly between tissue types and even within the same tissue as a function of disease, where the presence of various chemical and physical cues is known to influence cell division, migration, polarity, and metabolism. In particular, gradients of physical and chemical cues promote directed cell migration, a process that is essential during development, wound healing, immune responses and tumor progression (Jansen et al., 2015; SenGupta et al., 2021). For instance, during breast cancer invasion, high intra-tumoral stiffness, often due to increased deposition and crosslinking of collagen fibers, induces cancer cell migration into healthy, peripheral mammary tissue (Levental et al., 2009). Cells sense ECM architecture, such as aligned fibers, through a process termed contact guidance or topotaxis (Agudelo-Garcia et al., 2011; Nelson et al., 2014; Park et al., 2018). It is widely accepted that contact guidance provides a powerful cue to promote cancer invasion (Leclech and Villard, 2020). Aligned collagen fibers that radiate from the periphery of a primary tumor have been shown to promote directional migration in murine breast cancer models, and tumor ECM tension induces the alignment of random fibrillar matrices leading to greater directionality and invasive cell migration (Provenzano et al., 2006; Provenzano et al., 2008; Conklin et al., 2011).

In recent years, it has been demonstrated that many solid cancer cells principally invade local tissues by retaining their cell-cell contacts and migrating as collective groups of cells (Friedl and Gilmour, 2009; Cheung et al., 2013). Similarly, during wound healing, cell sheets polarize and migrate as a cohesive unit to close the wound (Mayor and Etienne-Manneville, 2016; Dekoninck and Blanpain, 2019). These collectively migrating cells integrate signals arising from cell-ECM interactions as well as cell-cell contact as they navigate complex ECM architectures. It is therefore critical that we understand the role of contact guidance during collective cell migration. To address these questions, we developed a method to investigate how multicellular groups undergo contact guidance using an adaptable *in vitro* system.

Wound healing assays, where a scratch “wound” is created in a confluent monolayer, are often used to study cell sheet migration but can create cell debris and be complicated by variations in “wound” size; in addition, this assay lacks physiologically relevant topology. As an alternative to wound healing assays, other groups have employed 2D spheroid migration assay (Deryugina and Bourdon, 1996; Vinci et al., 2012; Vinci et al., 2013; Labernadie et al., 2017). Previous studies have applied spheroids onto 2D surfaces to study durotaxis (McKenzie et al., 2018) and chemotaxis (Cuenca et al., 2020), yet how spheroids interpret contact guidance signals remains unexplored. Therefore, we developed a method to study collective cell migration dynamics using live cell imaging of spheroids on complex, fibrous 2D surfaces. The method allows visualization in phase and fluorescent modes when using cells expressing fluorescent probes. Furthermore, collection of the cells migrating on the 2D surfaces allows for concomitant biochemical and transcriptional characterizations of signaling pathways. By tuning of electrospinning parameters, we can investigate the influence of fiber diameter, fiber mat density, fiber alignment, and ECM adhesive components on collective cell migration behavior. Through this systematic deconstruction of the complex ECM, a better understanding of the mechanisms by which multicellular groups sense the various fibrous architectures of the ECM can be achieved and provide parameters that can be applied to more complex 3D models. To demonstrate the utility of this innovative system, we characterized collective migration of the highly invasive and metastatic breast cancer cell line MDA-MB-231 on aligned and random fiber mats.

## Methods

### Synthesizing electrospun fiber mats

To create fibrous 2D surfaces for studying cancer cell migration, we utilized electrospinning, a highly tunable fiber production method. Electrospinning has been previously established to generate fibrous topography from a variety of natural and synthetic polymers. In this method, charged polymeric material is ejected from a spinneret under a high-voltage electric field onto a collection surface where the material solidifies to retain a fibrous structure (Islam et al., 2019). Depending on the material type, the width of the fibers can range from nanometer to micrometer in scale and various crosslinking and functionalization methods can be sequentially employed to modulate the fiber mechanical properties or adhesive ligand presentation (Huang et al., 2003; Davidson et al., 2020; Depalma et al., 2021). The generation of fibrous topographies has been widely employed to model cell migration on fibrous ECM mimetics where properties such as fiber orientation, density, and ligand presentation can be readily tuned. For our experiments, we used fibers with 1–2 μm diameters, which mimic collagen fibers observed in the murine mammary gland *in vivo* (Conklin et al., 2011).

As originally published by Davidson et al. (Davidson et al., 2020), the synthetic fibers we used are composed of dextran vinyl sulfone (DexVS). DexVS matrices are resistant to hydrolytic degradation thereby allowing for long term cell culture experiments. The percent of vinyl sulfone functionalization of the dextran backbone was confirmed by nuclear magnetic resonance (NMR) spectroscopy. To create DexVS, we dissolved 5.0 g of high molecular weight (86 kDa) dextran in 0.1 N NaOH solution in a 500 mL bulb flask. In a chemical safety hood, the solution is stirred at approximately 250 rpm for 5 min or until the dextran is completely dissolved. Then, 12.5 mL pure divinyl sulfone is added to the bulb flask, which should turn the solution red within 1 min. The reaction is terminated by adding 2.5 mL 12 M HCl solution. If the solution does not turn pale green, we iteratively add 0.5 mL 12 M HCl solution. After terminating the reaction, the solution is transferred to 12–14 kDa dialysis tubing and dialyzed against Milli-Q for 72 h, changing the water every 12 h. After dialysis, 30 mL of purified DexVS solution is aliquoted into 50 mL conical tubes and frozen at −80°C for at least 1 h or until fully frozen. The frozen solution is lyophilized at −86°C and 0.040 mBar for 72 h. Finally, the 50 mL tubes are sealed with parafilm and the dry DexVS polymer is stored at −30°C until use.

To prepare the electrospinning solution, we thaw a 50 mL tube of dry DexVS at room temperature and weigh the dry polymer into a 20 mL scintillation vial. DexVS is dissolved at 0.6 g/mL in 1:1 dimethylformamide (DMF)/MQ with 10 mg/mL lithium phenyl-2,4,6- trimethylbenzoylphosphinate (LAP), 0.75 mM methacryloxyethyl thiocarbamoyl rhodamine B, and 5 vol% glycidyl methacrylate on a stir plate at ∼100 rpm for 2–4 h. The electrospinning solution can then be stored at 4°C for up to 1 month.

To create electrospun fibers mats, we draw DexVS electrospinning solution into a 1 mL syringe and attach a 305 mm 18G stainless steel (SS) needle. We seal the syringe-needle interface with electrical tape to prevent leakage and push out air bubbles through the needle before use. Next, we attach the syringe to an automated syringe pump at 0.2 mL/h flow rate and place it in a humidity-controlled glove box at 30%–35% relative humidity. To create random fiber mats, we place a round 12 mm^2^ glass coverslip on top of a grounded copper collection surface centered below the SS needle tip. Then we situate the tip of the SS needle 7 cm from the copper collection surface and connect the needle to the voltage source with an alligator clamp. We set the power source to −7.0 kV and turn on the automated syringe and voltage. As electrospinning solution begins to deposit, we adjust the copper stage to center the electrospinning cone above the glass coverslip. To create aligned fibers, we set the voltage source connected to the SS needle to +4.0 kV and balance the coverslip between two parallel electrodes set to −4.0 kV. We then deposit the electrospun fibers onto the coverslip for 30 s to 3 min depending on the desired thickness of the fiber mat. When the fiber mat reaches the desired thickness, we wet a cotton swab in water and run the swab tip around the edges of the coverslip to separate the fiber network on the coverslip from the surrounding copper collection surface. We then primary crosslink the fiber mats under 365 nm UV light at 100 mW/cm^2^ for 20 s to stabilize the fibers. The fiber-coated coverslips can be stored in a low humidity environment for up to 1 month.

The coverslips can be mounted on 24 well plates, thereby allowing the simultaneous recording of up to 24 different conditions. This is accomplished by drilling 10 mm^2^ holes in the bottom of a polystyrene 24 well plate using a Dremel 7,760 tool and a tungsten carbide, double-cut 25° pointed cone drill bit. After creating the well plate, we glue the coverslips on the plates using Dow SYLGARD™ 164 Silicone Elastomer kit, which is easily peeled off for further use after live cell imaging. After hydrating the fiber mat using heparin methacrylate (HepMA; see next section), we sterilize the coverslips with 70% ethanol for 10 min to prevent contamination. This setup can be easily scaled down to 12 or six well plate setups by spinning on larger coverslips.

### Live cell imaging of cancer spheroids

**FIGURE 1:**
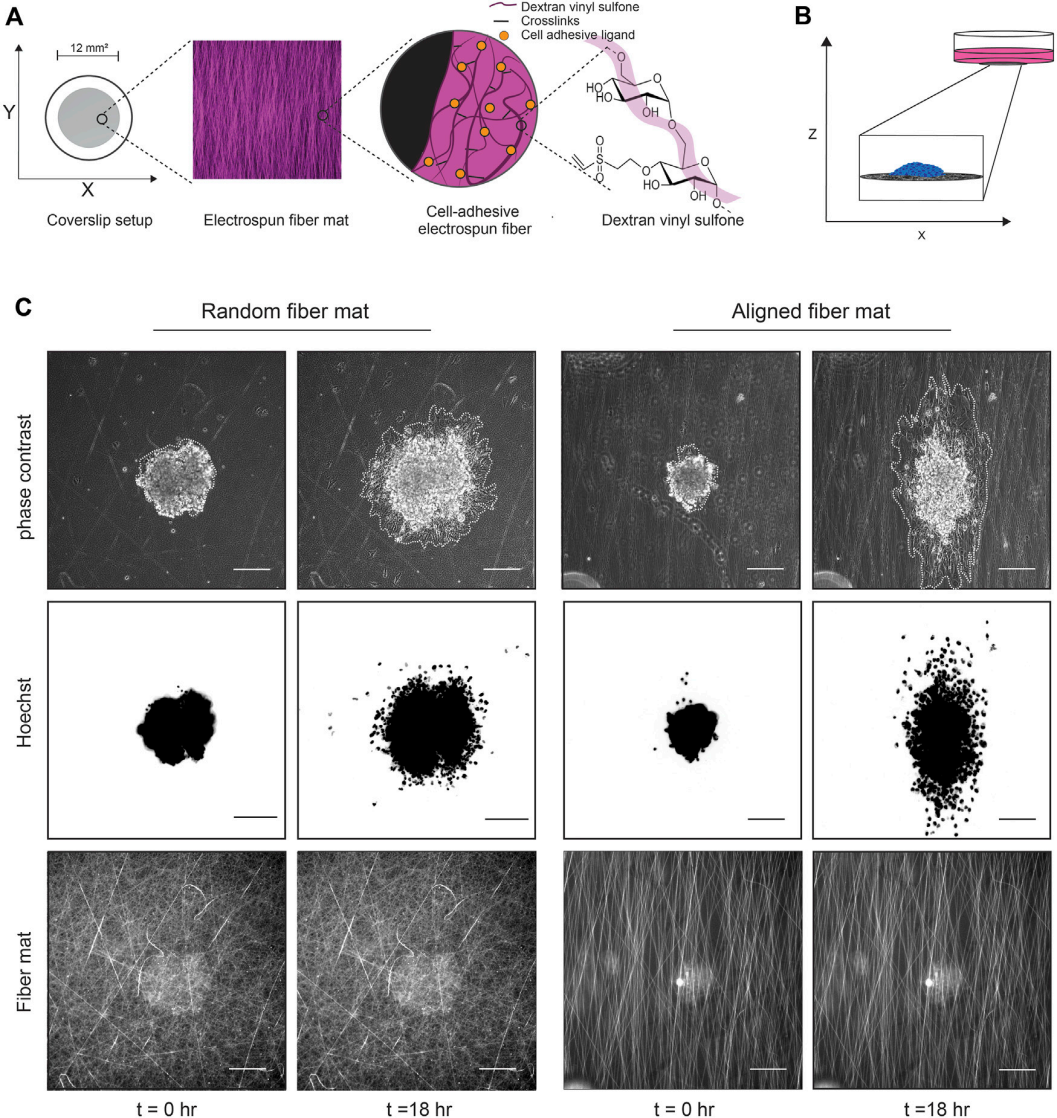
Experimental setup using DexVS fibers and cancer cell spheroids. **(A)** Cartoon depicting the method used to generate the fibers. Dextran is reacted with vinyl sulfone to create DexVS, which then is mixed with LAP and electrospun to create fiber mats on coverslips. **(B)** Cartoon depicting the 2D spheroid migration assay. After functionalization with collagen, cancer cell spheroids are plated onto fiber mats of interest and imaged over time to assess migration. **(C)** Representative phase contrast images of a MDA-MB-231 spheroid migrating on random or aligned fiber mats taken at the times 0 and 18 h. Spheroids are outlined in white to delineate the extent of cell spreading.

To allow cells to adhere to the synthetic fibers, we incubate the fiber mats in 50 μL of 2.5 w/v% HepMA, a structural analog to heparan sulfate, diluted in LAP followed by exposure to UV light (100 mW cm^−2^) for 20 s. Heparan sulfate proteoglycans (HSPGs), which bind many ECM proteins, are distributed throughout the ECM and are present at the cell surface (Bishop et al., 2007). The reaction covalently binds vinyl sulfone groups to methacrylate and enables coating of the fibers with any HSPG-binding proteins, such as type I collagen, fibrinogen, and fibronectin (Figure 1A). For our experiments we coat the fiber mats with 100 µg/mL type 1 bovine collagen for 1 h at 37°C. We also coat glass with 100 µg/mL type 1 bovine collagen for 1 h at 37°C as a control for our experiments. Following collagen binding, fiber mats or glass are rinsed three times with DPBS before adding spheroids.

To study collective cell migration, we use a spheroid migration assay where spheroids generated from the MDA-MB-231 cell line are plated onto fiber-coated 2D coverslips and imaged in real time as they migrate on the surface while retaining cell-cell contacts (Figure 1B). We generate spheroids using low adhesion 96 well plates coated with 1.2% 2-hydroxyethylmethylmethacrylate (polyHEMA). To create polyHEMA coated plates, we prepare a stock concentration of (12% or 120 mg/mL) polyHEMA solution and then dilute the stock to 1.2% in 95% ethanol. The stock and the working Poly-HEMA solutions can be stored at room temperature for several months. We then add 100 μL/well of 1.2% poly-HEMA and let the plates dry at 37°C until the alcohol is evaporated (2–3 days). To create spheroids, 1,000 cells in 80 μL are seeded into each well of the low adhesion V-bottom 96 well plates. After 6 days, a time point determined to generate tight spheroids with no necrotic core, we collect the spheroids and stain them with 0.8 μg/mL Hoechst 333482, a cell permeable nuclear dye, for 10 min at 37°C and wash in full media. For a 24 well plate setup, we seed ∼4 spheroids into each well in 500 μL of full media. The spheroids are allowed to settle and adhere to the fibers for 2 h at 37°C, 5% CO_2_ before live imaging^.^

The spheroids and fiber mats are imaged using a Zeiss Axio Observer Z.1 LED epifluorescence microscope equipped with an environment chamber that maintains temperature (37°C) and CO_2_ (5%) levels. The spheroids are imaged using phase-contrast microscopy every 10 min and fluorescence microscopy to image the Hoechst label every 30 min for 24 h (Figure 1C; Supplementary Movies S1, S2). In addition, an image of the fluorescent fiber mats on the first timepoint is acquired for later quantification of fiber mat density and alignment. After the experiments, the coverslips can be easily peeled off, and the well plate can be reused after sterilization with 70% ethanol.

## Results

### Utilizing TrackMate to generate cell tracks

**FIGURE 2:**
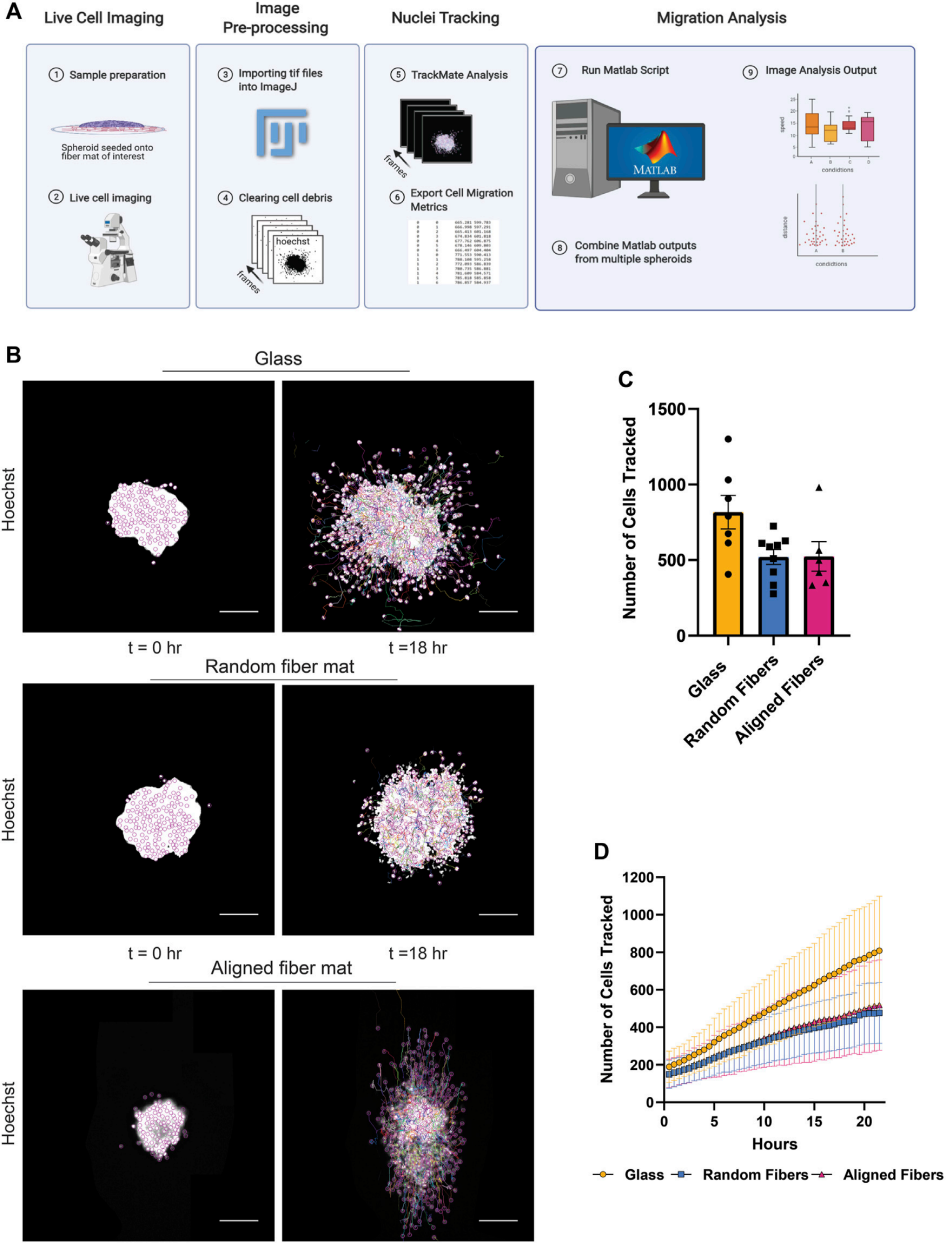
Methodology used to obtain cell migration metrics. **(A)** Workflow for image processing to compute cell migration metrics after live cell imaging. **(B)** Representative fluorescent images of a MDA-MB-231 spheroid migrating on glass, random or aligned fiber mats taken at the times 0 and 18 h. **(C)** Graph depicting the number of nuclei tracked at 22 h for spheroids migrating on the different surfaces. Each point represents an independent experiment. **(D)** Graph depicting the number of cells tracked as a function of time. Data represented as a mean ± SEM for **C** and^.^± SD for **(D)**. Figure 2A created using BioRender.com.

To obtain migration metrics, we use the open-source ImageJ plugin, TrackMate 6.0.2, to detect the Hoechst-positive nuclei and output individual nuclear locations over time (Tinevez et al., 2017). Before importing the live cell TIF file images into TrackMate, we remove non-migrating dead cells from the data set by selecting the nuclei with the polygon selection tool and clearing them using the clear function (Image → Clear). Next, we load the TIF files to track in TrackMate. For our analysis, we use a Laplacian of Gaussian (LoG) detector and a 12 μm diameter within TrackMate to detect nuclei. Next, we use the linear assignment problem tracker to link nuclei from frame to frame and create tracks. In the last prompt, the user can export the overlay of the tracks on the video and the spot statistics. The spot statistic data, which includes unique nuclei ID numbers, frame intervals, and the X and Y location of the nuclei, are then loaded into MATLAB for analysis. Using these values, we create an adaptable MATLAB script that calculates cell migration metrics, including Euclidean and accumulated distances, mean and median cell speed, instantaneous cell speed, mean cell directionality, and instantaneous cell directionality (Figure 2A). In addition, correlations between speed and directionality can be measured to further gain insight into migration phenotypes.

To demonstrate the capabilities of this platform, we measured the migration dynamics of the MDA- MB-231 breast cancer cell line on random and aligned electrospun fiber mats (Figure 2B; Supplementary Movie S3). Using the TrackMate plugin, we tracked 200–800 nuclei per frame (Figure 2C). We observed a gradual increase in the number of cells migrating over time due to persistent migration of the cells from the spheroid body (Figure 2D). Interestingly, we tracked a higher number of cells from spheroids after migration on glass compared to fiber mats, which is likely due an increase in cell speed on glass (see below).

### Analyzing cell migration metrics using MATLAB

**FIGURE 3:**
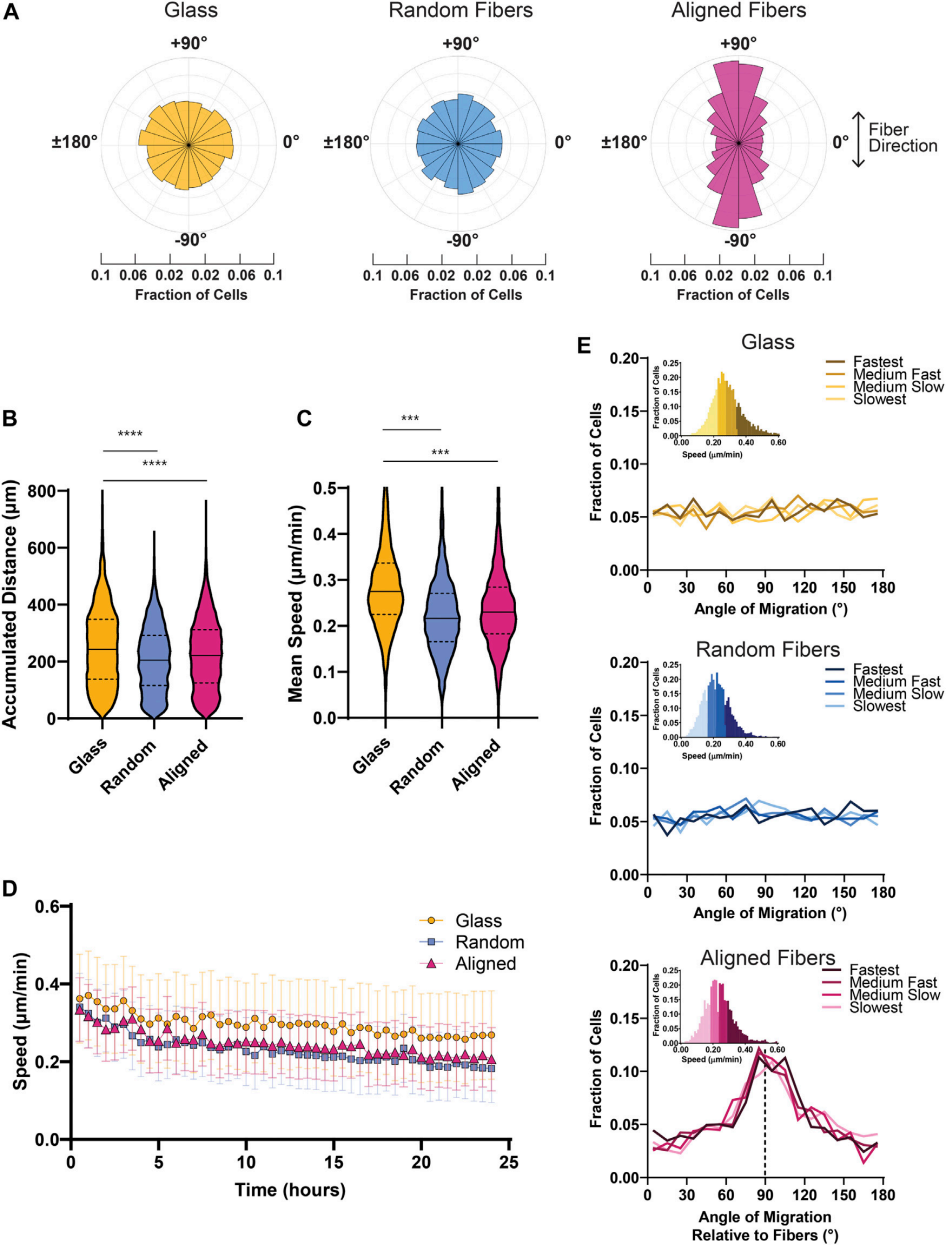
Bulk cell migration metrics of MDA-MB-231 spheroids migrating on glass and fiber surfaces. **(A)** Probability distribution plots of cell motion direction for MDA-MB-231 cells migrating on glass or fiber mats. **(B)** Graph depicting the mean accumulated distance for MDA-MB- 231 cells migrating for 22 h on glass, random fiber mats, or aligned fiber mats. **(C)** Graph depicting the mean cell speed of MDA-MB-231 cells migrating on glass or fiber mats. **(D)** Instantaneous cell speed (mean +/- SEM) of MDA-MB-231 spheroids over 24 h. **(E)** Graphs depicting the frequency of directional migration on glass or fiber mats. The different colors represent cell speeds that were binned as depicted. Analyses were performed on seven spheroids on glass, 11 spheroids on random fibers, and 11 spheroids on aligned fibers (*n* ≥ 3 biological replicates). *p* values are determined using an unpaired *t*-test. *p < 0.05, **p < 0.01, ***p < 0.001.

After analysis using our MATLAB script, we found that MDA-MB-231 spheroids show strong contact guidance when migrating on aligned fiber mats compared to isotropic surfaces such as glass and random fibers. The polar histograms illustrate the probability of migration direction with aligned fibers corresponding to the −90° and +90° directions (Figure 3A). We also found that cells migrate faster and farther on glass surfaces compared to fiber mats (Figures 3B, C). It has been reported that 3D collagen matrix alignment does not affect MDA-MB-231 cell speed (Riching et al., 2015), and similarly we found that in 2D the MDA-MB-231 cells migrate at comparable speeds on random and aligned fiber surfaces (Figure 3C). Additionally, we measured instantaneous metrics to assess migration changes over time. We found that speed decreased over time for all the conditions, but cells on glass showed a consistently higher cell speed at all time points (Figure 3D).

To dissect potential heterogeneity in migration within the group, we next determined whether cell speed correlated with cell directionality. To do so, we partitioned the cell speed data into four bins based on the individual mean cell speed of the cells and plotted the corresponding cell directionalities (Figure 3E). In the context of the contact guidance response observed on aligned fiber mats, we found no correlation between speed and directionality as cells in all bins exhibited strong directional migration on aligned fibers. In addition, we found that cells plated on surfaces without a directional cue–such as the random fiber mats or glass–migrated consistently in all directions. Taken together, these findings suggest that MDA-MB-231 cells display a highly uniform directional response to fiber alignment.

### Identification and quantification of single cell dispersion

We next set out to measure the migration metrics of cells within the group and those which break away from the group (i.e., single cells). To accomplish this, we used the k-nearest neighbor (KNN) algorithm, which compares a point of interest to candidate points using Euclidean distance. Using the *nearestneighbour* function (Brown, 2022), we compared the distance between each nucleus to all other nuclei within a frame, and identified nuclei that did not have nearest neighbors, based on a user defined input distance. After identifying the total number of single cells and their corresponding cell IDs within each frame, the code then loops through every frame, so the user has the number and cell ID of single cells in each timepoint. More specifically, we first loop through each frame and calculate X and Y coordinates for all nuclei. Next, using a nested loop, we assigned each nucleus as the point of interest and use the KNN algorithm to determine if that nucleus has neighbors within a specified distance threshold. After indexing through each nucleus in frame 1, the script then proceeds to frame 2. In addition to outputting the number of single cells in each frame and their corresponding cell ID, we utilized the *videowriter* function to output an AVI video file that displays all the cells over time and denotes “single” cells in color and “group” cells in grey. Finally, after classifying cells into “single” or “group” cells, cell speed and directionality between these two groups can easily be measured.

**FIGURE 4:**
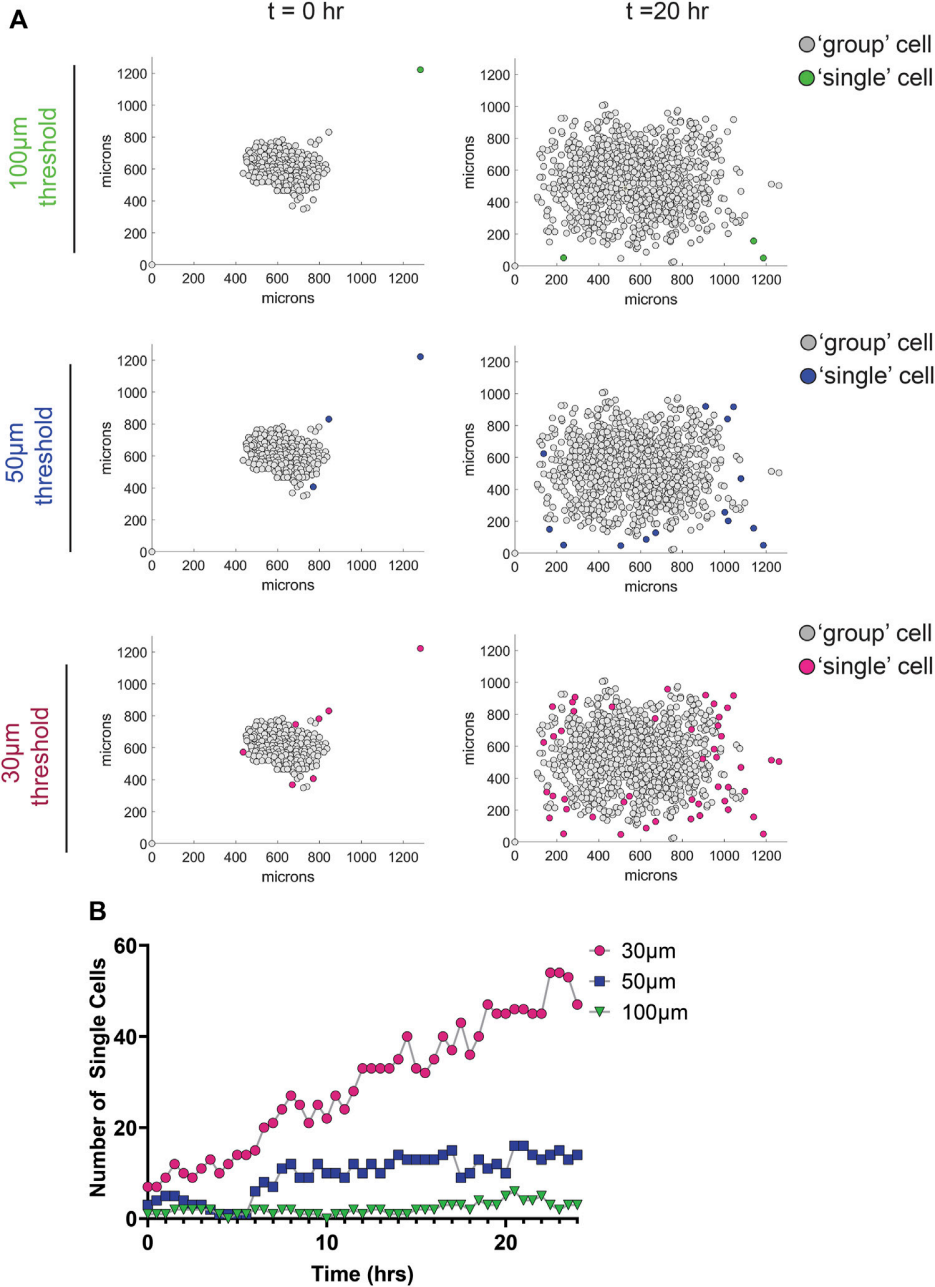
Effect of different nearest neighbor thresholds on single cell dispersion metrics of MDA-MB-231 spheroids migrating on glass. **(A)** Graphs depicting the location of cells 0 and 20 h after the initiation of migration using different nearest neighbor thresholds. Group cells are displayed in grey and single cells are displayed in color. **(B)** Graph depicting the number of single cells as a function of time for the different threshold used.

**FIGURE 5:**
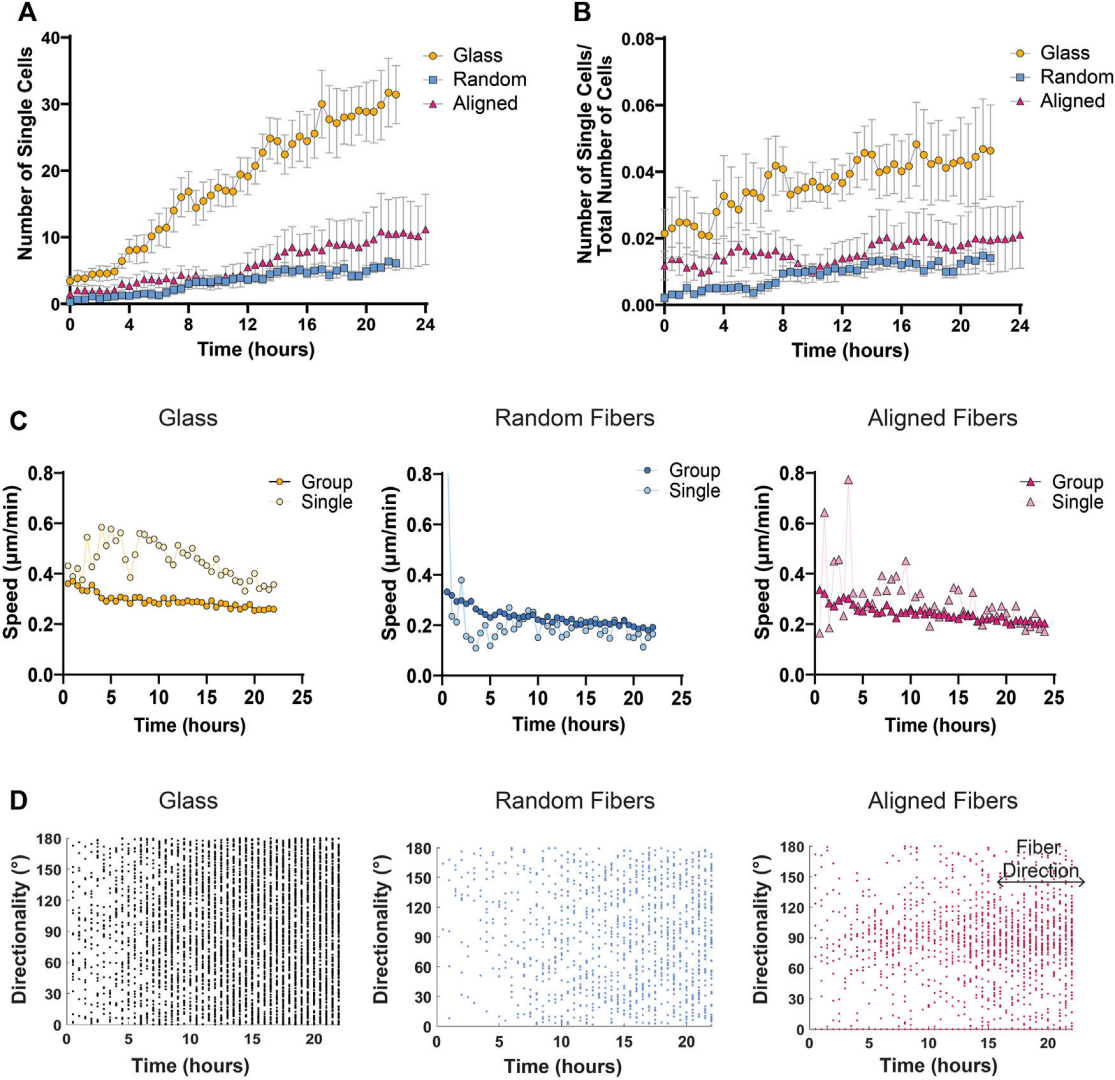
Single cell migration metrics of MDA-MB-231 spheroids migrating on glass and fiber surfaces. **(A)** The number of single cells detached from the cell sheet over time. **(B)** Differences in the number of single cells over the number of total cells to account for differences in total number of cells detected. **(C)** Mean cell speed of collective cell groups or single cells on various fiber mats over time. **(D)** Directionality of single cells over time on glass, random fiber mats, and aligned fiber mats with fibers at 90° direction for aligned fiber mats. Each dot represents the instantaneous directionality of one single cell. Analyses were performed on seven spheroids on glass, 11 spheroids on random fibers, and 11 spheroids on aligned fibers (*n* ≥ 3 biological replicates). All data presented as mean ± SEM.

To determine the optimal distance between nuclei for single cell designation in the MDA-MD-231 cells, we tested three KNN distance thresholds—30, 50, and 100 μm. As expected, we found that a higher threshold (100 μm) identified fewer single cells compared to a lower threshold of 50 or 30 μm (Figures 4A, B; Supplementary Movie S4). Using the AVI file exported from MATLAB and our original phase-contrast video, we then determined the best threshold for each cell line. By comparing the phase contrast video with the AVI file output from MATLAB, we were able to determine which nuclei belongs to single cells. We determined that a 40 μm threshold most accurately represents our data, which show that MDA-MB-231 cells migrating on glass coverslips display a significantly greater increase in single cells dissemination compared to cells on fibers (Figure 5A). In addition, to account for any variation between the total number of migrating cells, we plotted the ratio of the number of single cells over the total number of cells as a function of time on all three surfaces and found that the proportion of single cell dispersion also increases over time, and that spheroids on glass show the greatest proportion of single cell dispersal (Figure 5B). This loss of group migration on glass, which also correlated with increased speeds, is likely due to the lack of topographical complexity.

In addition to identifying and quantifying the number of single versus groups of cells over time, we further investigated any changes in speed or directionality between these two groups. By comparing the mean cell speed over time of cells within groups versus single cells on fiber or glass surfaces, we found that single cells move faster than cells within the group at all time points on glass surfaces, while both single and group cells on fiber mats tend to move at the same speed over time (Figure 5C). Based on previous literature, we envision that the lack of substrate complexity and increased substrate stiffness of glass surfaces allow for more stable cell-substrate interactions leading to increased traction force and cell speed (Pelham and Wang, 1997; Lo et al., 2000; Chan and Odde, 2008; Rens and Merks, 2020). Finally, we also studied the directionality of single cells by plotting each cell’s instantaneous directionality over time. Not surprisingly, we found that single cells migrating on aligned fiber mats exhibit strong contact guidance compared to single cells migrating on glass or random fibers surfaces (Figure 5D).

### Visualizing protein localization and protein expression changes during migration on fiber mats

**FIGURE 6:**
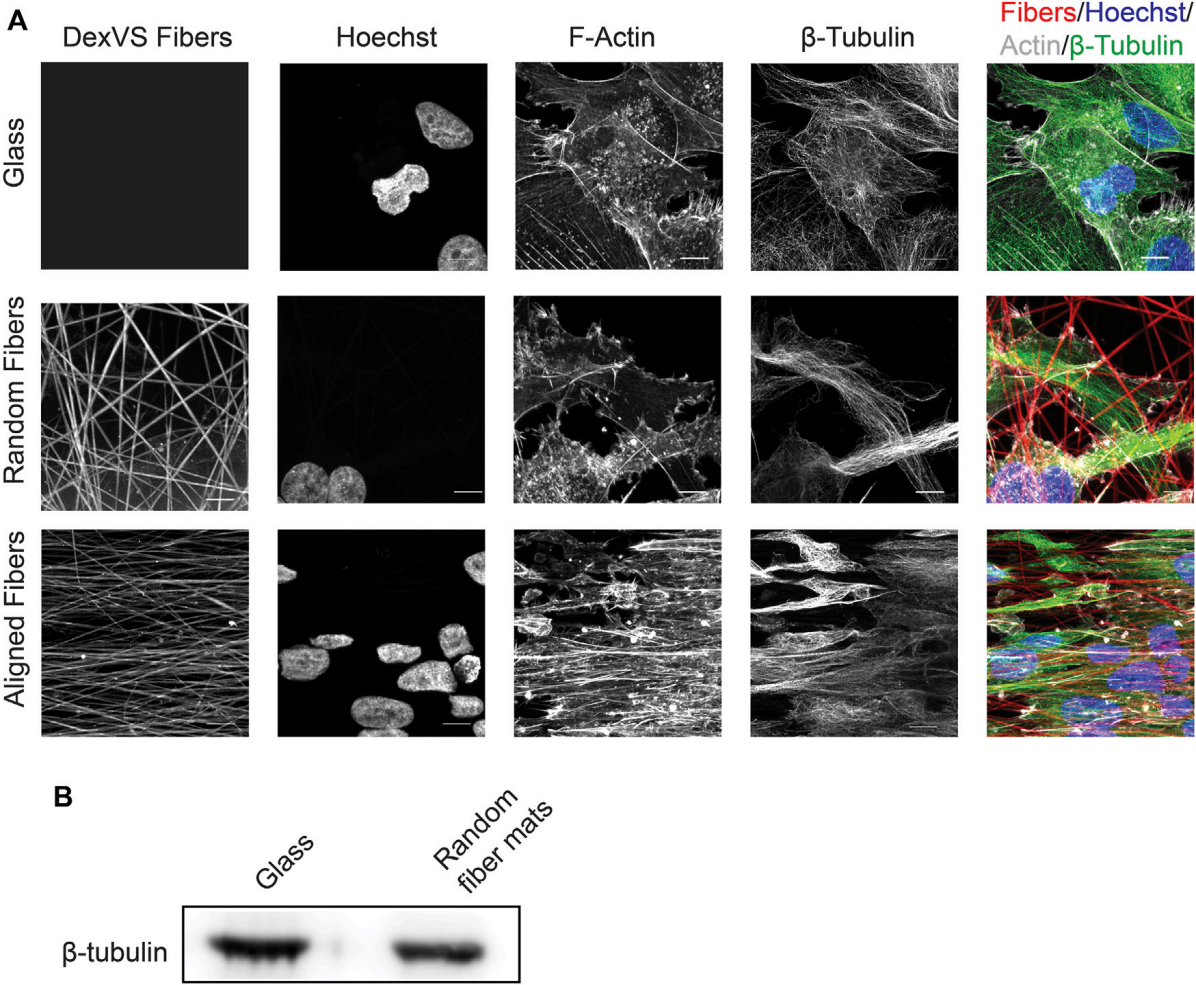
Expression and distribution of β-tubulin after cell migration on glass or fiber mats. **(A)** Immunofluorescence localization of β-tubulin and F-actin after 24 h of cell migration on fiber mats or glass. Scale bar is 10 μm. **(B)** Immunoblot depicting β-tubulin expression after 6 days of cell migration on glass of random fiber mats.

The dynamic distribution of proteins of interest in cells migrating on the various substrate can readily be studied by constructing cells expressing fluorescently tagged proteins and capturing images at relevant time points during migration (see live cell imaging section above). In addition, immunofluorescence can be used to visualize static protein distribution in cells migrating on the various surfaces. For both applications, the fluorescent fibers provide a mean to assess the distribution of protein of interests in relationship with the fibers (Figure 6A). Furthermore, the fixed samples can be complimentary to the live cell imaging analyses by using cells expressing fusions with fluorescent markers. As a proof of concept, we fixed cells that migrated on the surfaces for 24 h with 4% PFA for 10 min at 37°C followed by washing with PBS. Next, we permeabilized the cells in 0.1% Triton-X100, and blocked non-specific binding using 4% bovine serum albumin (BSA). We stained for β-tubulin at 4°C overnight in 1% BSA. After conjugation with a compatible fluorescent secondary antibody and incubation with phalloidin-488 and Hoechst, the cells were imaged using a Zeiss 880 confocal microscope. As expected, MDA-MB-231 cells showed a dramatic difference in cytoskeletal morphology on random, aligned or glass surfaces (Figure 6A). On glass surfaces, the cells commonly displayed stress fibers due to the rigidity of the glass, and β-tubulin fibers radiated from the center of the cell. On random fibers, the cytoskeleton formed strong interactions with the random matrix that seemed to direct F-actin and β-tubulin localization. Whereas MDA-MB-231 cells migrating on aligned fibers showed an elongated and aligned actin and microtubule cytoskeleton.

In addition to assessing migration dynamics using live cell imaging, the system we developed can readily be used to measure changes in protein expression and/or activation status after collective cell migration on the fibrous surfaces using western blot analysis. For these applications, we prepare cell lysates once all the cells have migrated out of the spheroids onto the 2D surfaces, which is 6 days for the MDA-MB-231 cells. To obtain enough material for these measurements, we plate approximately ten spheroids onto a 25 mm^2^ coverslip. To extract proteins of interest, we place the coverslips on ice and use 200 μL of 2X Laemmli lysis buffer per coverslip to initiate cell lysis. After 5 min on ice, the lysates are scraped, transferred in a microcentrifuge tube and centrifuged at 10,000×g for 15 min at 4°C to remove fibers and clarify the cell lysate. At this point, the samples can be subjected to gel electrophoresis and changes in a protein of interest can be investigated using immunoblotting. As an example, we collected sufficient protein to compare differences between β-tubulin expression in MDA-MB-231 cell migration on random fibers or glass coverslips (Figure 6B).

## Discussion

In summary, we present a highly adaptable method to investigate collective cell migration on complex surfaces using live cell imaging, in depth automated migration quantification, as well as biochemical analyses which can be performed following live cell imaging. Building on previous systems investigating cell migration in response to chemical and stiffness cues, we developed a method to provide a holistic understanding of how different cell types migrate on fibrous surfaces of various topographies and allow the investigation of the molecular changes that occur as cells encounter and migrate on these surfaces. We demonstrate that by combining the use of cancer cell spheroids with electrospun fiber mats, it is possible to measure migration dynamics of collective groups as well as individual cells moving on physiologically relevant fiber topographies. As a proof of concept, we demonstrate the full capabilities of our method using the highly metastatic breast epithelial cell line, MDA-MB-231, on two distinct fiber topographies, using glass as a control.

In addition to optimizing the spheroid-fiber migration assay, we also successfully automated much of the cell migration analysis using the TrackMate plugin from ImageJ (Tinevez et al., 2017) and by developing a custom MATLAB script. Our MATLAB script can quickly process all datasets within a user-specified folder allowing for rapid migration analysis. In addition to calculating bulk migration data, we also utilize the k-nearest neighbor algorithm to distinguish single versus group migration, allowing us to analyze migration changes within these subsets. We showed that MDA-MB-231 cells show an increased mean cell speed and accumulated distance on glass surfaces compared to fiber mats. Additionally, we showed that speed and directionality are not correlated in MDA-MD-231 cell migration. We further investigated differences in migration between cells within the group and cells which break away from the group and found that while single cells migrating on fiber mats show similar speeds as their corresponding group cells, MDA-MB-231 single cells migrate much faster on glass than cells that are part of the group.

The assays can easily be modified according to the user’s interests: changing cell matrix protein (i.e., fibronectin, collagen IV); including other cell types to produce a co-culture system; genetic manipulations or inhibitor treatment; as well as changes in fiber size, density and organization or fiber mat size. In addition, developing methods to expand the capabilities of the cell tracking workflow to incorporate more intricate quantification such as changes in nuclear morphology over time can readily be done. Finally, in the future, we envision that this system could be scaled to 3D using a fiber-enforced hydrogel (Matera et al., 2019; Su et al., 2021). While image acquisition and quantification methods are more challenging in 3D environments, we recently published an automated image analysis framework that can be used in future 3D experiments (Hiraki et al., 2022). Additionally, we are currently expanding our 2D aligned fiber mat system to a more complex 3D aligned fiber system through the use of superparamagnetic iron oxide nanoparticles (SPIONs) embedded in synthetic fiber segments (Hiraki et al., 2021).

Our approach has some limitations that leave room for future improvement. These include the lack of automation during the TrackMate analysis, which we plan to streamline by integrating TrackMate with MATLAB using the ImageJ-MATLAB extension. The addition of this automated step will greatly increase the efficiency and speed of the workflow. Also, while k-nearest neighbor identification of single cells allows for an equal classification based on a user-defined constant between experimental conditions, there are drawbacks to this method. For example, the user is required to define the distance threshold which subsequently determines the classification of a single or group cell. So, while this adds bias to the quantification, it also allows for analysis of cell types with different migration phenotypes and cytoskeletal morphologies. In the future, it may be useful to stain cells with a cytosolic fluorescent dye, so that this classification can be done without user bias.

## Data Availability

The raw data supporting the conclusions of this article will be made available by the authors, without undue reservation. The code developed for this work is available upon request.

## Supplementary material

Supplementary Movie S1 MDA-MB-231 spheroid migrating on random mats. Images were acquired every30 min for 24 hrs and played at 10 frames per sec.

Supplementary Movie S2 MDA-MB-231 spheroid migrating aligned fiber mats. Images were acquired every 30 min for 24 hrs and played at 10 frames per sec.

Supplementary Movie S3 MDA-MB-231 spheroid migrating on glass (left), random (middle) or aligned (right) fiber mats. Images were acquired every 30 min for 24 hrs and played at 10 frames per sec. Nuclei are outlined in magenta and tracks are shown using multicolored lines.

Supplementary Movie S4 Movies depicting the location of MDA-MB-231 cells migrating on glass for 24 hrs and played at 5 frames per sec. Group cells are displayed in light grey and single cells are displayed in either green (100 μm threshold), blue (50 μm threshold), or magenta (30 μm threshold). The consequence of using different nearest neighbor thresholds is presented.
